# Ecological assessment of extreme temperature and fine particulate matter (pm2.5) impact on diabetes service and outcomes in Thailand

**DOI:** 10.1186/s12889-025-24003-5

**Published:** 2025-08-15

**Authors:** Bumi Herman, Jason Kai Wei Lee, Xihao Du, Yot Terawattananon

**Affiliations:** 1 Present Address: School of Global Health, Faculty of Medicine, Chulalongkorn University, Bangkok, Thailand; 2 Heat Resilience and Performance Centre, Yong Loo Lin School of Medicine, National University of Singapore, Kent Ridge, Singapore; 3 Human Potential Translational Research Programme, Yong Loo Lin School of Medicine, National University of Singapore, Kent Ridge, Singapore; 4 Department of Physiology, Yong Loo Lin School of Medicine, National University of Singapore, Kent Ridge, Singapore; 5 Department of Epidemiology and Biostatistics, School of Public Health, Shanghai Jiao Tong University School of Medicine, Shanghai, China; 6 Health Intervention and Technology Assessment Program (HITAP), Ministry of Public Health, Bangkok, Thailand

**Keywords:** Diabetes screening, Maximum temperature, Air pollution, Blood glucose control, Health system, Thailand, Climate change

## Abstract

**Background:**

Environmental changes, such as extreme heat and air pollution, are assumed to exacerbate chronic conditions like diabetes and increase demand for related health services. This study investigates how daily maximum temperature and air pollution affect diabetes service utilization in Thailand.

**Method:**

Using ecological analysis, national diabetes service data from the Thai Ministry of Public Health and ERA5 LAND satellite data (from 2018 to 2023) were examined. The data included 2-meter ambient temperature and particulate matter (PM2.5) concentrations, adjusted for health system variables, aggregated risk factors, the COVID-19 outbreak response, and spatiotemporal correlations. A generalized additive mixed model (GAMM) was employed to assess the effects of maximum temperature and PM2.5 on diabetes screening and the proportion of patients with controlled blood glucose.

**Results:**

The analysis revealed an annual average maximum temperature of 38.6 °C and an average PM2.5 concentration of 21.6 µg/m³. National diabetes screening was 87% of the target, and only 30% of diagnosed patients achieved the recommended blood glucose levels. Maximum temperature was found to non-linearly reduce diabetes screening, particularly between 31.6 °C and 41.9 °C, while no significant association was observed with PM2.5 levels. Temperatures above 32.7 °C were associated with lower proportions of patients achieving recommended blood sugar levels, while lower annual PM2.5 levels (16.4 to 18.8 µg/m³) had similar benefits.

**Conclusion and recommendations:**

Extreme heat was associated with reduced diabetes screening, while PM2.5 showed no significant effect. These findings highlight the need to adapt health service delivery to reduce the impact of heat stress on service utilization. Strategies such as shifting screening services closer to communities, promoting telemedicine, and strengthening the role of primary care and village health volunteers may help maintain access to care during periods of extreme temperature.

**Supplementary Information:**

The online version contains supplementary material available at 10.1186/s12889-025-24003-5.

## Introduction

Diabetes Mellitus (DM) is a condition caused by impaired insulin function, leading to elevated blood glucose levels and long-term debilitating complications. In 2024, around 589 million people globally were living with DM, with the majority residing in low- and middle-income countries. In 2024, diabetes directly caused 3.4 million deaths. Moreover, 42.7% of adults living with diabetes were undiagnosed [1]. In Thailand, it was estimated that 6.4 million people were living with diabetes in 2024, with approximately 33% of adult age 20–79 years remaining undiagnosed [1].

The World Health Organization emphasizes the importance of early intervention in diabetes, advocating for behaviour modification and enhanced screening to maintain blood glucose levels within recommended ranges through the STEPwise Approach to Surveillance **(**STEPS) approach [2]. Thailand’s National Service Plan for Non-communicable Diseases (NCDs) requires ongoing evaluation, particularly with consideration of regional sociodemographic factors. Recognizing spatial variations in risk factors is crucial for developing more effective, decentralized health management strategies [3].

Several studies have highlighted the impact of climate change on general health, including exposure to heat and air pollution [4]. Research in Denmark found that a single day of heat exposure ≥ 28 °C can trigger early physiological responses, particularly in the cardiovascular, pulmonary, and renal systems [5]. Additionally, studies emphasize the link between extreme ambient heat exposure and increased hospital admissions and emergency department visits related to diabetes [6, 7]. In Thailand, the average annual temperature in 2023 was 27.51 °C [8]. With a more robust model to estimate global warming, the world will still face an increasing temperature for the next decades [9], which become a threat to diabetes management.

Particulate matter (PM2.5), small particles found in the air, can infiltrate the distal respiratory tract and induce toxicity, leading to airway inflammation and disrupting immune homeostasis [10]. Exposure to PM2.5 also raises levels of tumour necrosis factor-alpha, interleukin 6 (IL-6), resistin, and leptin, potentially contributing to insulin resistance and the onset of Type 2 DM [11]. In 2019, ambient air pollution, particularly PM2.5, was responsible for an attributable mortality rate of 104 per 100,000 people globally. While Thailand’s rate of attributable mortality was lower than its neighbouring countries (46.5), the country experiences seasonal surges in PM2.5, especially during the agricultural season. In 2020, Thailand recorded a mean annual PM2.5exposure of 31 µg/m³, exceeding the recommended standards [12]. Moreover, a global systematic review highlighted the significant impact of long-term PM2.5 exposure on the development of diabetes [13].

It is hypothesized that the impact of climate change on diabetes may also be mediated by disruptions to diabetes services. Extreme temperatures [14] and polluted air can hinder outdoor activity, which may affect individuals’ ability to access healthcare services, including diabetes screenings and treatments. The general population may be less likely to attend screenings due to the challenges posed by heat and poor air quality, as well as difficulties accessing treatment at healthcare facilities. However, studies to confirm this hypothesis are lacking, and further research is needed to understand if this is occurring in Thailand.

This study hypothesizes that, when adjusted for other factors (including health system elements, percentage of high-risk population and presence of Coronavirus Disease 2019/COVID-19) outbreak), maximum temperature and PM2.5 levels may influence diabetes screening uptake and treatment outcomes. Therefore, it is essential to implement changes in diabetes healthcare services to make them more resilient to climate change. This study will also explore potential solutions for adapting diabetes services to environmental challenges, particularly in Thailand.

## Methodology

### Study design and source of information

Given the complex pathways linking environmental factors to health outcomes, this study adopts an ecological design to focus on health system resilience and equity rather than individual-level biological mechanisms. By analyzing spatially aggregated data, we aim to illustrate how climate change impacts diabetes outcomes through disruptions in healthcare services, offering actionable insights for targeted interventions. This approach complements mechanistic studies by providing a service-oriented, policy-relevant perspective essential for climate-health adaptation and system-level responses. This study drawing on data from 2018 to 2023 obtained from the Health Data Center (operating under Thailand’s Ministry of Public Health), the Pollution Control Department, and the National Statistics Office of Thailand. The dataset is organized by Thailand’s administrative divisions: provinces, districts, or health regions, and is available at annual, quarterly, or monthly intervals.

### Study population

This study focuses on 22,834,784 targeted populations above 35 years old, of which 6,776,280 underwent screening and 3,585,443 registered patients under ICD E.10–14, which covers type 1, type 2, other specified and unspecified diabetes mellitus. The criterion of “above 35 years old” was based on the data availability and relevance to the study objective. The dashboard used for this analysis does not include registry data for individuals under 35 years old, and access to this specific subgroup was not granted. Furthermore, individuals under 35 years are more likely to have type 1 diabetes, which is primarily driven by non-modifiable genetic and autoimmune factors, differing from type 2 diabetes and other types, where modifiable risk and external factors and external exposure to environmental hazards play a significant role. Given the constraints in identifying individuals within the electronic dataset, obtaining explicit consent for data use was not required.

### Variables

This study collected data on demographic factors, health service infrastructure, and geospatial indicators. Demographic variables included sex ratio, reflecting its association with diabetes mellitus [15]. Health service infrastructure data comprised health insurance coverage, accredited community hospitals offering NCD services, the rate of healthcare personnel qualified for diabetes screening (doctors, dentists, and nurses), and village health volunteers per 1000 population. Aggregate data from the health dashboard on high-risk behaviours,alcohol consumption, Body Mass Index (BMI) over 25 kg/m² for the general population and diabetes patients, and active smokers were included, given their strong links to metabolic syndrome and diabetes [16]. Continuous treatment is defined as the percentage of people registered and received diabetes treatment. Geospatial factors, such as topography, population density, and urban-rural distinctions, were also considered, assuming their potential influence on physical activity levels [17], body weight, and diabetes progression.

The environmental factors examined in this study include the annual average maximum temperature and ambient PM2.5 levels. Using Google Earth Engine (GEE), the study calculated the maximum 2-meter temperature for each province in Thailand based on the ERA5-Land hourly temperature dataset. The 2-meter temperature reflects the temperature perceived by humans, as opposed to land surface temperature, which can be significantly higher due to surface effects, especially in urban settings. Notably, in Turkey, the ERA5/ERA5-Land dataset has shown no significant difference compared to station-based trends [18].

The ambient annual average PM2.5 concentration (in µg/m³) was sourced from the Pollution Control Department and university data centres (including Chulalongkorn University CUSense, Yakkaw Sensor Mae Fah Luang University and Chiangmai University Climate Change Data Center, which publicly shared to Air Quality Index China Network/AQICN). The analysis focused on the main centroid of each main station. For one province with missing data, values were interpolated using inverse distance weighted (IDW) interpolation, with distances calculated using the Haversine formula [19].

d = 2r × arcsin( √( sin²(Δφ/2) + cos(φ1) × cos(φ2) × sin²(Δλ/2) ) )

Where:

d = Distance between the two points (in kilometers or meters).

r = Radius of the Earth (mean radius ≈ 6,371 km).

φ1, φ2 = Latitudes of point 1 and point 2 (in radians).

Δφ = φ2 - φ1 (difference in latitude in radians).

Δλ = λ2 - λ1 (difference in longitude in radians).

The level of COVID-19 outbreak control was defined as the ability to reduce the percentage of new infections among the screened population to below 5% within 28 days of new cases emerging in a given area. This study also incorporates spatial analysis to account for spatial autocorrelation and identify cluster distributions of parameters and outcomes.

### Specific outcomes

The study outcomes include the percentage of diabetes screenings and the percentage of individuals with controlled blood glucose levels. Diabetes screening is defined as the number of individuals aged 35 years and older who received screening relative to the target population. The second outcome is defined as the number of individuals achieving blood glucose levels within the recommended target range relative to the total number of patients.

### Potential bias

This study’s ecological design limits inferences about individual-level interactions between independent and dependent variables. The count of cases relies on the number of individuals screened, but data collection was restricted to those aged 35 and older, excluding assessments of Type 1 diabetes, which typically manifests earlier.

PM2.5 data availability was a challenge, as not all regions provided comprehensive observations. In addition to data from non-governmental monitoring stations (particularly from universities, interpolation was used, though interpolated values may not fully reflect actual pollution levels. Provinces with multiple air quality stations focused on those near city centres, potentially overlooking rural disparities. For temperature data, while satellite measurements show similar reliability in another country, namely Brazil, India, and United States [20], a small analysis should assess interrater agreement between satellite and station data.

### Quantification and discretization

COVID outbreak control is defined as a binary response, where level 1 indicates a controlled situation throughout the year, and level 0 indicates any uncontrolled situation during 2020–2022. The pre- and post-COVID pandemic periods will be coded as 1. Other variables are treated as previously described.

### Statistical analysis

The study began with descriptive statistics and data visualization for the parameters. Bivariate analysis was then performed for each parameter in relation to the outcome. A Generalized Additive Mixed Model (GAMM) was employed to examine the relationship between various predictors and the response variables. We selected Generalized Additive Mixed Models (GAMMs) over other models including time-series or spatial model for their ability to flexibly model non-linear exposure-response relationships, account for spatial autocorrelation, handle repeated measures over time, and address overdispersion in count data, providing a robust and interpretable framework suitable for our ecological analysis of environmental impacts on diabetes service utilization. The model is depicted in the following equation:

1 $$\begin{aligned} Outcome\;=&\beta0\;+\;\beta1(variable\;1)\;+\;\beta2(\;variable\;2)+\;\dots\dots\;\\&+\;f(variable\;3)\;+\;\;f(lat,long)\;\\&+\;f(PM\;2.5)\;+\;f(max\;temp)\;+\dots\dots.\;+\;\epsilon \end{aligned}$$

The equation consists of linear terms and smooth terms particularly for environmental parameters to capture potential nonlinear relationships with the response variable. This formula fixes the smooth term to be a penalized spline with a predefined, fixed basis function. ` lat`, and `long` represent the latitude and longitude of the provinces. The model is fitted with a quasi-poisson distribution family, suitable for count data with overdispersion,

Potential correlations within the data were accounted for by incorporating a random intercept term for provinces, addressing within-subject correlations over time, which is essential in longitudinal or repeated measures studies. After fitting the model, the results were summarized to assess the estimated coefficients, their significance, and other relevant statistics. Predictors were trimmed using a hierarchical procedure, and the adjusted R-squared criterion was applied to select the best model.

The predicted values of the smooth variables and its corresponding standard errors were extracted from the fitted model. The Relative Risk (RR) was then calculated by exponentiating the smooth fit values. To quantify the uncertainty around the RR estimates, 95% confidence intervals were computed by adjusting the smooth fit values with the standard errors. These confidence intervals were used to assess the precision of the RR estimates. Finally, the RR values along with their confidence intervals were incorporated into the prediction dataset for further analysis and visualization using R program.

## Results

**Fig. 1:**
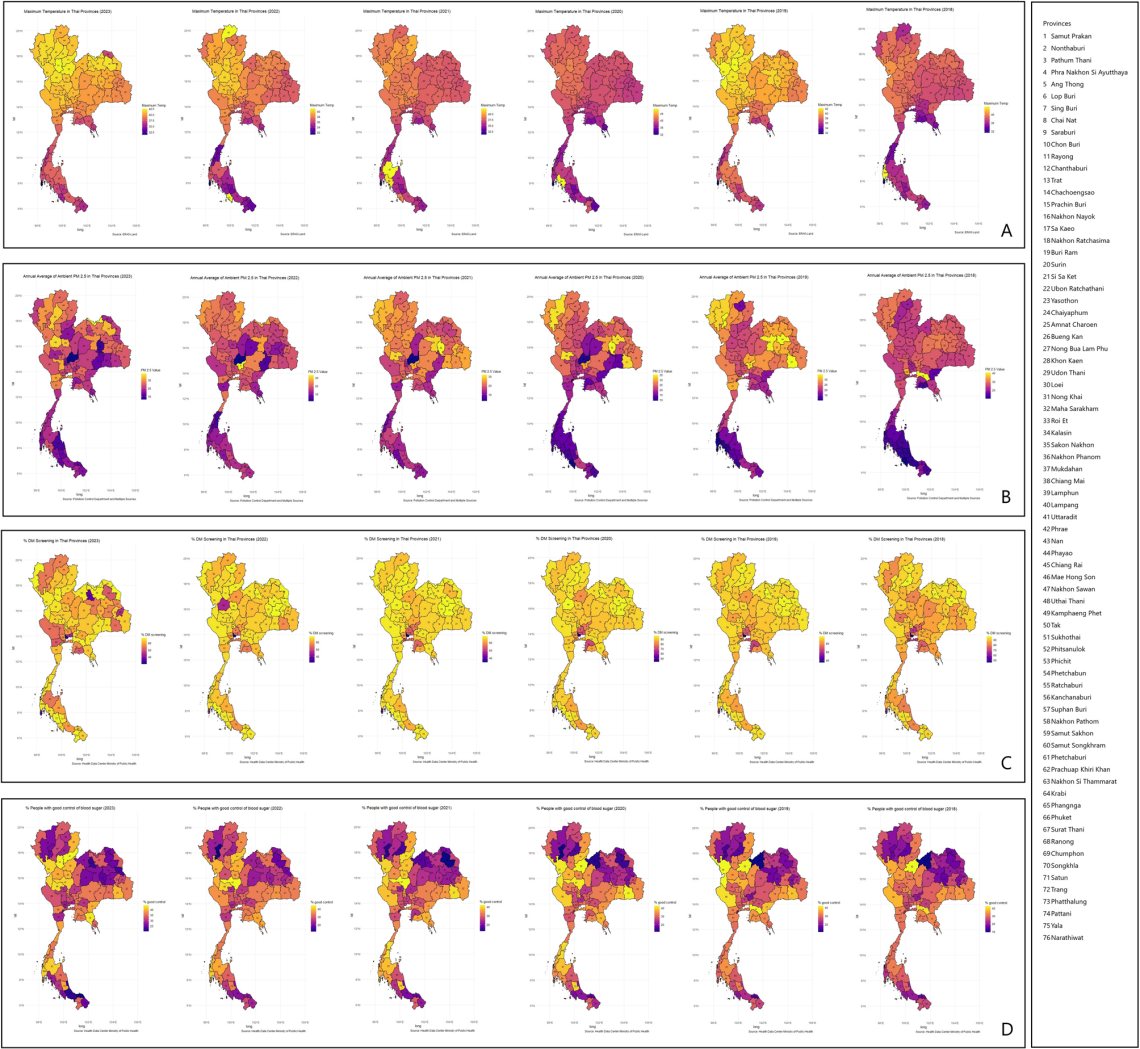
Trend of environment parameters and diabetes services

From 2018 to 2023, the average maximum temperature in Thailand reached 38.6 °C. A sudden decrease in the maximum temperature was observed in 2020, followed by a gradual increase until 2023. Interestingly, the northern part of Thailand, characterised by higher altitudes, recorded a higher annual average maximum temperature than the southern and central regions (Fig. 1).

Regarding air pollution, the annual average ambient PM2.5 concentration was 21.6 µg/m³, which is below the WHO Interim Target-2 standard of 25 µg/m³ that was used as Thailand’s Pollution Control Department standard prior to 2023 [21]. Like the maximum temperature, the northern region experienced consistently higher PM2.5 levels throughout the year, while the southern region was comparatively less polluted (Fig. 1b).

The five-year average screening rate for diabetes (DM) among the 35-year-old and older population was 87%, although some areas in central Thailand, predominantly urban settings, recorded lower screening percentages (Fig. 1c) (Bangkok was excluded due to missing data on the national dashboard). Only 60% of the population could access continuous DM care. In terms of the percentage of people achieving good blood glucose control, lower rates were observed in the northern, northeastern, and southern regions, as well as in areas near the capital (Fig. 1 ).

**Table 1:** Values of variables from 2018–2023

Variables	Average	Min-Max	*p*-value
Maximum Temperature in °C	38.58 ± 2.09	31.20-46.81	< 0.001
Sex Ratio	0.93 ± 0.04	0.79–1.03	< 0.001
% Screened per target population (> 35 years old)	87.01 ± 11.01	20.17–97.47	< 0.001
% patient received continuous treatment	60.12 ± 14.99	22.08–90.28	< 0.001
% patient achieved good blood glucose	29.82 ± 6.91	9.29–44.47	< 0.001
% Accredited Health Provider	87.14 ± 17.82	0-100	0.237
% people above 15–59 years old with higher BMI	47.88 ± 5.46	31.63–57.53	0.07
% Active Smoker (> 15 years old)	7.58 ± 3.63	0.80 −19.61	< 0.001
Rate of Health Personnel capable to perform Blood Tests/1000 population	4.09 ± 1.84	0.80-16.13	< 0.001
% coverage of health insurance	91.69 ± 7.39	55.28–99.82	< 0.001
Rate of Health Volunteers/1000 population	14.88 ± 5.82	1.79–31.15	0.307
% Alcohol Drinker (> 15 years old)	10.69 ± 7.62	0.54–55.95	0.048
Annual Average of PM Value in µg/m³	21.61 ± 6.30	1.90–40.70	< 0.001

Table 1 presents the average values of predictors and highlights the significant differences in means across the years. In general, the Thai population has more women as represented by a Sex ratio of less than 1. It is important to note that less than one-third of diabetes patients can achieve the recommended blood glucose level. In terms of the proportion of high-risk individuals, around 48% of the population had a BMI over 25 kg/m2. The Thai population proportion who actively engaged in alcohol drinking was higher than those who actively smoked. In terms of the health system element, no differences were observed in the Rate of Health Volunteers per 1,000 population or the percentage of Accredited Health Providers over the six years. Combining the rate of doctors, nurses and dentists who are capable of performing blood glucose tests, there were 4 health personnel per 1000 population. In general, Thai people are covered by three types of national health insurance, however, around 8–9% of the population not covered by health insurance were mainly foreigners and those with unclear health insurance protection (of which less than 0.5% of the total population).

**Table 2:** Annual changes of variables from 2018–2023

Variables	2018	2019	2020	2021	2022	2023
Maximum Temperature in °C	37.84	39.39	39.32	37.73	37.57	39.63
% Screened per target population (> 35 years old)	87.69	88.64	90.03	88.74	86.68	80.31
% patient achieved good blood glucose	27.17	28.60	29.83	29.62	30.44	33.29
Annual Average of PM Value in µg/m³	24.18	24.15	21.52	19.64	17.81	22.40

Table 2 summarizes annual trends from 2018 to 2023. Maximum temperature peaked in 2019 and 2023. Screening rates remained high until 2021 but declined in 2023. The proportion of patients with good blood glucose control steadily improved each year. PM2.5 levels generally declined until 2022, with a slight increase in 2023. Further correlation reveals an inverse relationship between maximum temperature and screening (*r* = −0.31, *p* = 0.55), also with between PM 2.5 and screening (*r* = −0.04, *p* = 0.94) and glucose control (*r*=−0.35, *p* = 0.49). However, maximum temperature and glucose control has positive correlation (*r* = 0.42, *p* = 0.40) which should be interpreted cautiously.

### Diabetes services models

Following the hierarchical regression and model comparison with diagnostic metrics, Table 3 presents the factors affecting diabetes screening (Model 1) and good control of blood glucose levels (Model 2). Health insurance coverage was associated with diabetes screening, with a positive estimate, indicating that a higher proportion of people covered by insurance contributes to increased diabetes screening. There were no significant associations between other fixed parameters and diabetes screening. 

**Table 3:** Generalized additive mixed models of diabetes services parameters

Variables	MODEL 1			MODEL 2		
	Estimate	Std. Error	Pr(>|t|)	Estimate	Std. Error	Pr(>|t|)
(Intercept)	4.537	0.239	< 0.001	3.045	0.112	< 0.001
% Continuous Treatment				0.005	0.001	< 0.001
DM Incidence/100,000 population				− 1.1e-05	0.000	0.887
% DM patients with higher BMI				1.5e-04	0.002	0.925
% of Health Insurance Coverage	0.003	0.001	0.008			
Health Personnel Rate	0.001	0.005	0.867			
COVID Control Outbreak	−0.014	0.021	0.519			
Sex Ratio	−0.336	0.218	0.125			
Smooth Parameters	edf	Ref.df	p-value	edf	Ref.df	p-value
Maximum Temperature	9.000	9.000	0.017	9.000	9.000	0.330
PM2.5 in µg/m³	9.000	9.000	0.930	9.000	9.000	0.312
Health Volunteer Rate	9.000	9.000	< 0.001			
Proportion of People with higher BMI	9.000	9.000	< 0.001			
Proportion of Alcohol Drinker	9.000	9.000	< 0.001			
Proportion of Active Smokers	9.000	9.000	< 0.001			
% Accredited Health Center	9.000	9.000	0.019	9.000	9.000	0.053
Spatial variability	8.231	12.460	0.060	27.180	29.330	< 0.001
Adjusted R-Square/Deviance Explained	0.648/72.8%			0.594/62.3%		

Regarding the smooth parameters, significant non-linear associations were observed between maximum temperature, health volunteer rate, the proportion of the population with higher BMI, alcohol consumption, active smoking, and the proportion of accredited health providers with diabetes screening.

Further interaction analysis shows significant interaction between health insurance coverage and both maximum temperature (*p* < 0.001) and PM2.5 (*p* = 0.04), where areas with higher insurance coverage (above median) experienced a decline in screening rates as temperatures increased. In contrast, in areas with lower insurance coverage (at or below the 25th percentile), screening rates increased with rising temperatures. Moreover, the interaction between PM2.5 and health insurance coverage was significantly associated with diabetes screening rates. All quartiles of health insurance coverage showed a slight increase in diabetes screening as PM2.5 levels rose. Notably, the lowest quartile exhibited a sharp relative increase, although the overall screening rate remained lower than those in higher quartiles (supplementary Table 1).

**Fig. 2:**
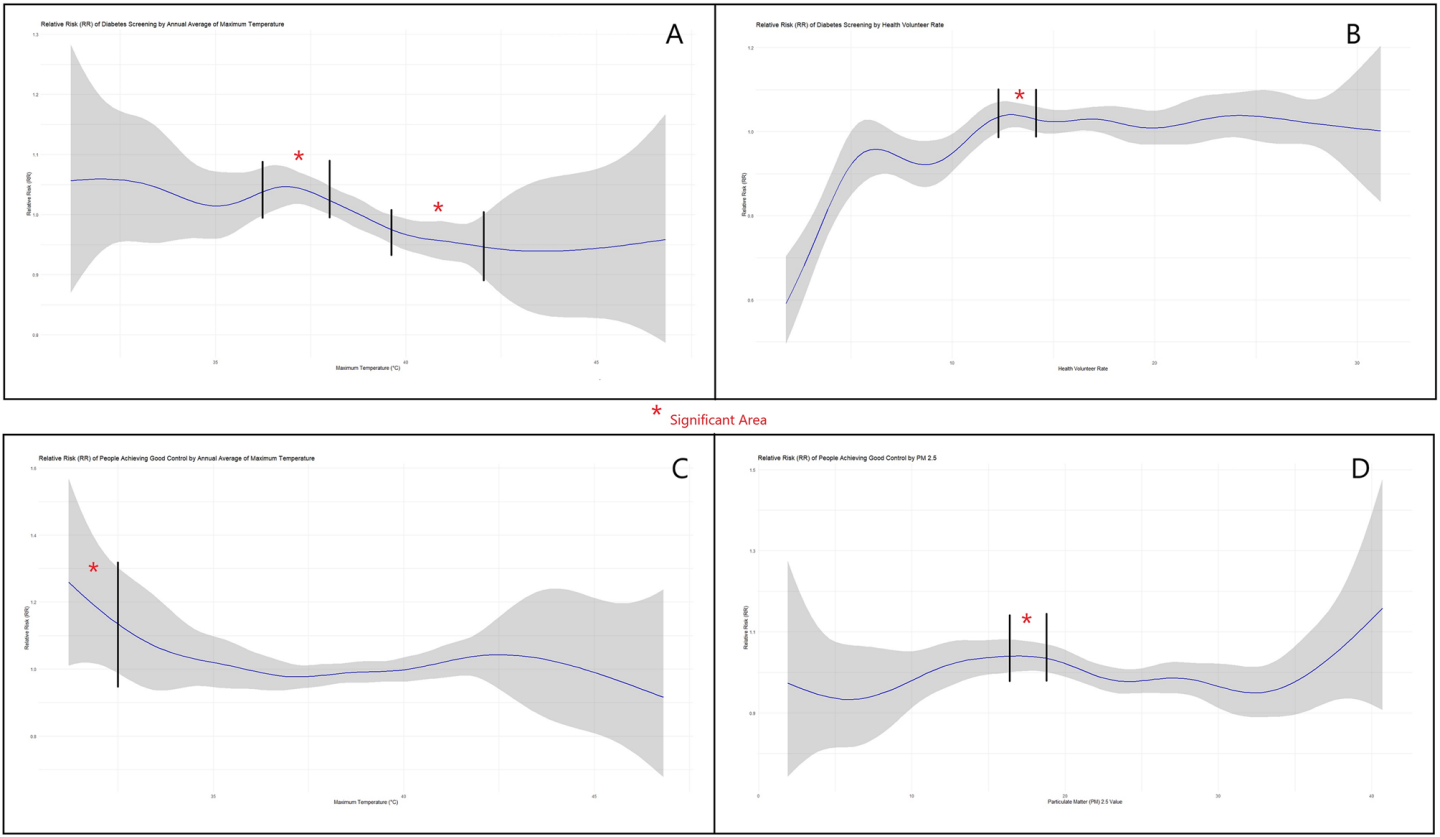
Smooth parameters of Model 1 and 2

The interaction between PM2.5 and the percentage of accredited health centres revealed a different trend: areas with a lower percentage of accredited centres experienced decreased screening rates as PM2.5 levels increased, while areas in the upper percentile showed a positive trend. However, this difference was not statistically significant. (*p* = 0.06) (Supplementary Table 1) (Fig. 2).

 Associations are presented in relative risk. Figure A depicts the association between diabetes screening and maximum temperature. Figure B explains the association between the rate of screening and health volunteers. Figure C illustrates the non-linear association between maximum temperature and the proportion of people with good control of blood glucose. Figure D demonstrates the association between the proportion of people with controlled blood glucose and PM2.5.

A decline in the relative risk of diabetes screening indicates that higher maximum temperatures are associated with lower diabetes screening rates seen in Fig. 2A. Specifically, maximum temperatures between 39.5 °C and 41.9 °C significantly reduced the percentage of diabetes screening. Conversely, lower temperatures, below the average mentioned in Table 1, were associated with higher diabetes screening rates, particularly in the range of 36.4 °C to 37.8 °C. Regarding PM2.5, no significant effect was found on diabetes screening (Supplementary Fig. 1).

This study also estimates the ideal number of health volunteers for diabetes screening. The association reveals a ceiling effect between the health volunteer rate and diabetes screening. The minimum ideal rate for a significant increase in diabetes screening was between 12.5 and 13.9 volunteers per 1,000 population as seen in Fig. 2B.

Regarding the percentage of people who achieved good blood glucose control (defined by reduction of Hba1c to 7 without comorbidity or according to personalised regimen among those with comorbidities and assessed by physician), Model 2 in Table 3 demonstrates a significant positive association with the proportion of patients receiving continuous treatment. An increase in diabetes incidence was related to a lower proportion of people achieving good blood glucose levels, although this relationship was not statistically significant. There was a positive, non-significant association with the percentage of diabetes patients with higher BMI. Other smooth parameters did not show any significant non-linear associations.

Maximum temperatures below 32.7 °C were associated with a significantly higher proportion of people achieving the recommended blood sugar level as seen in Fig. 2C. However, in Fig. 2D, there was a mixed effect with annual PM2.5 levels between 16.4 and 18.7 µg/m³, significantly associated with a higher proportion of people achieving the recommended blood glucose level.

This study also reveals a significant spatial correlation regarding the proportion of people achieving the recommended blood glucose level. Figure 1 d highlights clusters of areas with lower proportions. In the southern part of Thailand, lower values were observed near the Thai-Malaysian border, as well as in northern provinces and areas near the Thai-Lao border.

## Discussion

### Climate, coverage, and care diabetes screening in Thailand

This study highlights that Thailand experiences higher annual average maximum temperatures (after Singapore and Cambodia) [22] and ambient PM2.5 levels compared to other ASEAN countries [23]. While the impacts of climate change and environmental degradation on health services remain underexplored, this research reveals non-linear effects on diabetes services, particularly screening and blood glucose control. These findings underscore the urgent need to mitigate climate change by slowing temperature rises, controlling ambient air pollution, particularly PM2.5, and strengthening health system resilience to address diabetes.

Between 2018 and 2023, the annual diabetes screening rate among the population aged 35 and older averaged 87%, a significant improvement from 67% a decade ago [24]. However, this still falls short of the 90% target. Lower screening rates were observed in areas near the capital and, more recently, in parts of the northeast.

Disparities in socioeconomic status remain a key barrier to diabetes screening, as demonstrated in Singapore [25]. Thailand’s universal health coverage (UHC) reduces financial hardship caused by out-of-pocket payments for screening, particularly for older and economically disadvantaged populations [26]. In this study, health insurance coverage was associated with higher screening uptake (Table 1).

Thailand’s UHC system is funded primarily through taxation and consists of three main schemes: the Civil Service Medical Benefits Scheme, the Social Security Scheme, and the Universal Coverage Scheme. These schemes collectively ensure diabetes screening for the majority of the population aged 35 and older [27]. However, certain groups—such as foreigners, individuals who pay out-of-pocket, and those with unclear registry status—remain uninsured and face higher risks of undiagnosed diabetes and financial hardship from unexpected healthcare costs. Challenges such as procedural complexities and the unaffordability of private insurance mirror findings in the U.S [28].

Although this study could not directly compare screening rates between insured and uninsured populations due to data limitations, even insured individuals may hesitate to undergo screening. This hesitancy is often driven by concerns over the costs of managing complications if diagnosed. For instance, individuals are more likely to undergo screening for complications when covered by insurance [29]. Integrating comprehensive diabetes care—including screening and complication prevention—under UHC could improve overall screening rates.

### Socio-demographic and behavioral factors in diabetes screening

No significant associations were found between diabetes screening rates and health personnel ratios, outbreak control measures, or sex ratios. However, non-linear relationships were observed between screening uptake and specific high-risk population characteristics. For instance, screening rates were significantly higher when the prevalence of active smokers ranged between 7.1% and 13.9%, but lower when it was below 3.8% (Supplementary Fig. 2). Similarly, a higher proportion of individuals with obesity (BMI > 30) was associated with lower screening uptake. A comparable trend was noted for alcohol consumption, with screening rates higher when alcohol prevalence was below 2.8%.

These trends suggest altered perceptions of risk and benefits among these groups, including fear of poor diagnoses or concerns about adherence to treatment plans. Such behavioral barriers require further investigation. This is concerning, as these groups are at heightened risk of undiagnosed diabetes. For example, a study in Peru found a positive association between diabetes screening uptake and obesity prevalence [30]. Similarly, reducing obesity prevalence by just 5% could prevent approximately 13,000 new diabetes cases [31].

### Core findings: environmental factors and screening uptake

Our study found that higher maximum temperatures significantly reduced diabetes screening rates, likely by affecting time use and limiting mobility, especially among vulnerable populations to screening facilities. These findings are supported by previous research on the impact of heat on human mobility and commuting patterns [32]. To examine this assumption, we used the available Google Community Mobility data for Thailand from January to October 2022 [33]. We extracted mobility trends for specific places, including public places, pharmacies and public transport, during the study period and compared them with temperature and PM2.5 levels. (Details are provided in the supplementary Table 2.) The analysis showed that mobility to these places tended to be lower when temperature (*r* = −0.43) and PM2.5 levels (*r* = −0.40) were higher, although these correlations were not statistically significant.

No significant association was observed between ambient PM2.5 levels and diabetes screening uptake. While exposure to PM2.5 is linked to Type 2 diabetes incidence [34] Increasing PM2.5 levels should ideally drive higher screening rates to identify undiagnosed cases. However, the supplementary figure shows non-significant reductions in screening uptake at PM2.5 levels below 7.8 µg/m³, an increase above 36 µg/m³, and unexpectedly lower rates in the range of 26.2–35.6 µg/m³. These patterns may reflect behavioural responses to daily PM2.5 fluctuations, such as reduced outdoor movement that discourages healthcare visits. However, this remains speculative, as the available dataset does not include high-resolution daily PM2.5 measurements necessary to explore these dynamics in depth. From a statistical standpoint, while the model allowed for a flexible relationship using the maximum Estimated Degree of Freedom (edf) set by the software, the high p-value (0.93) indicates that observed patterns may result from random variation rather than a meaningful association. This highlights the need for cautious interpretation, as the smooth term may be overfitting noise in the data. More refined analyses using daily or short-term PM2.5 data could help clarify these trends.

Supporting this assumption, a study found that everyday mobility is influenced by air pollution levels [35] as demonstrated in supplementary Table 2. However, further robust analysis using daily PM2.5 data is necessary to confirm this, although the nature of the dataset currently limits such analysis. Decentralizing screenings closer to populations could reduce individual PM2.5 exposure during commutes [36], particularly going to healthcare facilities. Additionally, attention should be given to indoor air pollution exposure in public transport systems, including vehicles [37] and underground stations.

Longer commutes further exacerbated heat exposure risks, particularly in areas with poorly designed public transport systems [38]. People with diabetes also face higher risks of acute complications—such as diabetic ketoacidosis, hyperosmolar hyperglycemic state, and hypoglycemia—requiring hospitalization during heat exposure [39].

### Health system interaction with environmental factors

In supplementary Table, the significant interaction between environmental parameters—particularly maximum temperature and PM2.5—and health insurance coverage reveals a complex relationship between environmental stressors and health service utilization. Interestingly, in areas with higher health insurance coverage, we observed a decline in diabetes screening rates as temperature increased. This finding may reflect system-level barriers or behavioural adaptations under environmental stress. For instance, in well-covered areas, individuals may have more healthcare options and thus delay or avoid routine services during heatwaves (as assumed by the common pattern of human mobility during extreme heat [32]), expecting to return later when conditions improve. Healthcare providers also experienced more workload during heat waves [40]. Hence, health services in these areas might also adjust service hours, staffing, or outreach during such periods, inadvertently reducing access.

In terms of PM2.5, the interaction results in our study provide intriguing results. A study in China found that short-term exposure to air pollution reduced the outpatient visits, but in the longer term, it increased the visits due to the adverse effects of continuous exposure for more than 15 lag days [41]. Given the nature of our data, which records the annual average PM2.5 levels, the slight increase in diabetes screening rates—particularly in areas with lower health insurance coverage—may be attributed to perceived greater vulnerability due to socioeconomic disparities (e.g., higher baseline risk, poorer living conditions), targeted outreach during pollution events, increased health awareness prompting care-seeking, and a catch-up effect among previously underserved populations. These hypotheses, however, warrant further investigation.

The observed interaction between PM2.5 and accredited health centres suggests that areas with more accredited health centres may respond better to rising PM2.5 levels by increasing diabetes screening, possibly due to better resources, trust, and outreach capacity. In contrast, areas with fewer accredited centres may lack the infrastructure to support such a response. A systematic review highlighted the responsiveness of accredited primary care centres in uncertain situations [42].

### Adaptation and resilience strategies

To enhance climate resilience, several strategies should be considered. First, geographical targeting of areas with high PM2.5 exposure and elevated temperatures is essential. With current real-time monitoring technologies, developing such maps is feasible. A study in Northern California successfully applied geospatial mapping to identify pre-diabetes hotspots using demographic and socioeconomic data [43]. Expanding such models to include environmental factors such as PM2.5 and temperature could further improve the identification of undiagnosed cases, enabling more effective and efficient intervention planning.

Following hotspot identification, clear resource allocation and tailored policy interventions are necessary. These may include the deployment of skilled health workers, distribution of screening kits, enhanced training for community health volunteers, and mobile screening units that can be mobilized during periods of high PM2.5 levels or extreme heat. Previous assessments of health workforce distribution in Thailand have shown equitable allocation since 2016, although minor discrepancies remain across provinces with different economic levels [44]. Therefore, focusing on capacity building for existing health workers is considered more appropriate than prioritizing workforce quantity.

### Community and home-based interventions

Would decentralizing diabetes prevention and early detection efforts to community and home-based services help address challenges posed by environmental changes, such as extreme heat and air pollution? Active screening efforts, such as leveraging community health workers [45] or facilitating self-testing [46]could mitigate these challenges. This study identified an optimal health volunteer ratio—12.5 to 13.9 per 1,000 population—to support screening efforts. Rather than increasing volunteer numbers, improving their capacity to deliver diabetes services is critical.

Deploying test kits to households within the UHC framework is another viable option, though challenges related to cost, efficacy, and the availability of self-testing kits [47] must be addressed. A review stated that temperature may affect the storage of the test kit and, therefore, affect the accuracy of blood glucose readings. This could also apply to the storing of medication and insulin as well [48].

Advancements in health data utilization and risk prediction, such as ADA-based scoring systems (a scoring system that estimates an individual’s risk of diabetes using factors like age, BMI, family history, and lifestyle, where a higher score indicates a higher risk of diabetes), could offer less invasive methods for identifying at-risk individuals [49].

Decentralizing testing to nearby community health providers is another promising approach when test-kit deployment is not feasible. In the study in the United States, involving community health workers (CHWs) on screening, particularly during COVID pandemic resulting in higher screening rates [45]. This involvement also gained significant screening and referral outcomes in a large study in India [50]. With adequate training, CHWs in Thailand could provide screening and refer cases to physicians. Given their large numbers—up to one million—this approach could be an effective way to scale up screening efforts while reducing unnecessary travel and exposure to extreme heat.

Referral-based screening programs involving community pharmacies and general practitioners have shown effectiveness in the United States [51]. Expanding pharmacists’ roles to conduct screenings within Thailand’s UHC framework could minimise the need for travel, thereby reducing heat exposure risks. A systematic review found that pharmacists play a significant role in improving medication adherence and clinical outcomes through both in-person and remote pharmacy services [52]. In Thailand, reviews of pharmacist roles and capacity highlight the public’s positive perception of pharmacists as reliable health professionals for counseling and medication use guidance. However, there is also recognition that Thai pharmacists could take on a greater role in primary healthcare, provided they receive additional knowledge and training [53]. In Australia community pharmacy diabetes screening trial, over 90% of participants reported high satisfaction and lifestyle improvements, with those receiving point-of-care tests being twice as likely to follow up with their GP. General practitioners also viewed pharmacist referrals as appropriate, suggesting that community pharmacy screening can effectively support T2DM prevention [54] and feasible to be conducted in Thailand.

### Policy and infrastructure recommendations

While the following measures may not directly target diabetes services, they play a crucial role in mitigating the broader health impacts of environmental factors. Raising public awareness about the connection between environmental exposures and chronic diseases is essential. To drive effective behavior change, communication strategies should be theory-based and evidence-driven, incorporating localized, positively framed messages, actionable advice, and an emphasis on co-benefits such as improved health and climate outcomes [55]. In addition to enforcing existing air quality standards for PM2.5 and other pollutants in Thailand, it is important to introduce temperature-related indicators like the heat index, excess heat factor, and the number of consecutive hot days to better inform the public. Implementing a health warning system—such as Germany’s model—can also help identify and protect vulnerable populations [56].

Investing in public infrastructure is critical. For example, providing cooling shelters in public spaces or affordable air filtration systems aligns with the Climate-Health-Infrastructure-Pathways (CHIP) Model [57]. Thailand has made efforts through Sustainable Total Resource Management (STRM) in the healthcare sector to balance sustainability goals with operational costs. However, progress has been slow due to unclear policy direction and weak enforcement [58]. A pilot program for climate-resilient hospitals in selected provinces demonstrated a multi-pronged approach: strengthening infrastructure (e.g., ventilation and cooling systems, green energy), enhancing preparedness and surveillance through early warning systems and emergency plans, and training healthcare workers on climate-related health issues [59]. Notable examples can be seen in hospitals in Bangkok and Chiang Mai, especially in their efforts to monitor and respond to air pollution.

Human resources remain a challenge. Health workers have reported inadequate training, insufficient resources, and poor interdepartmental coordination [60], and this also occurred in Thailand [61]. Targeted training should cover climate risk assessment, emergency preparedness for extreme weather events, early warning systems and disease surveillance, energy-efficient operations, and managing medications and equipment under harsh conditions. Health workers also need to be equipped to educate and engage the public on climate-health issues.

Thailand has taken a step forward by shifting the burden of non-communicable diseases (NCDs) to primary care and community-based efforts, thereby reducing pressure on advanced healthcare settings. However, to sustain this progress, clear policy direction, adequate resource allocation, and technical support are necessary. Additionally, there should be a greater emphasis on mapping obesogenic environments [62] as mentioned previously.

### Improving service outcomes and addressing treatment inequality

From Model 2, continuous treatment emerged as the only factor significantly associated with a higher percentage of individuals achieving recommended blood glucose levels. While an increase in diabetes incidence was associated with a lower proportion of controlled patients, this relationship was not statistically significant. Achieving optimal blood glucose levels requires continuous treatment, especially within the first six months after initiating therapy. This should be complemented by consistent self-monitoring to prevent adverse treatment effects. A six-month subscription-based SMBG intervention package in Malaysia demonstrated improved blood glucose control, as reflected in HbA1c levels, and enhanced health-related quality of life [63].

Regarding environmental factors, Fig. 2 shows a significantly higher proportion of individuals achieving recommended blood glucose levels at lower maximum temperatures and PM2.5 levels ranging between 16.4 and 18.7 µg/m³. This may be due to more favorable weather conditions, enabling better access to healthcare facilities for treatment. It is important to highlight the significant spatial autocorrelation of this parameter, which indicates the presence of specific clusters. Areas with lower rates of controlled patients are often located at higher altitudes and in rural regions, consistent with findings from Peru [64]. This suggests that geographical challenges and disparities in healthcare services contribute to the issue. A study in India identified key barriers to patient-centred diabetes care in rural areas, including resource shortages, knowledge gaps, and motivational disparity [65].

There is an assumption that higher temperatures may affect clinical outcomes due to improper storage of medications, particularly insulin [66] and inaccurate monitoring caused by device malfunction at elevated room temperatures, as previously discussed. A study revealed that the majority of diabetes patients treated with insulin in Thailand did not store their insulin at the correct temperature. Approximately 60% of patients stored it in a refrigerator, assuming that lower temperatures would better preserve the insulin, while 40% kept it at room temperature, which often exceeded 30 degrees Celsius [67]. Although there was no significant effect of maximum temperature on blood glucose level, it is still essential for health workers to communicate the impact of higher temperature on their testing and medication storage.

Telemedicine has been proven to reduce travel and increase overall productivity gains in an Australian study of outpatient consults [68]. Reduction of travel and commuting could be translated as lower emissions and lower risk of heat exposure. A review also demonstrated the reduction of HbA1c and complications from the telemedicine service for diabetes [69]. However, shifting diabetes care toward telemedicine and telepharmacy still presents challenges, even though the WHO advocates for the digitalization of NCD services [70]. Additionally, comprehensive examinations at well-equipped health facilities are often necessary to evaluate treatment efficacy, which complicates the implementation of remote care for NCDs.

### Strength and limitations

This study integrates climate factors as key variables influencing diabetes services, addressing the need for health systems to become more adaptive to inevitable climate changes. By utilizing robust models and drawing insights from a national database, the study also accounted for unexpected events, such as the COVID-19 outbreak, which may have affected healthcare services.

Several limitations should be considered. First, the study collected data from different age groups, as the diabetes screening was targeted at individuals aged 35 years and older, while other indicators were gathered for individuals aged 15 years and above. This discrepancy arises from the eligibility criteria for diabetes services under Universal Health Coverage (UHC), which may not extend to younger populations for screening. The cut-off age of 35 years is based on historical evidence suggesting a higher prevalence of Type 2 diabetes in middle-aged and older adults, which informed national screening guidelines aimed at maximizing cost-effectiveness and resource allocation. However, this age threshold may overlook emerging trends in diabetes epidemiology. A 2022 national diabetes registry in Thailand revealed that among patients under 30 years of age, 62.6% had Type 1 diabetes and 30.7% had Type 2 diabetes. Notably, 61% of those with Type 2 diabetes had onset between the ages of 20 and 29 [71], This trend is driven by lifestyle changes, rising obesity rates, sedentary behavior, and poor dietary habits among adolescents and young adults, suggesting that lowering the screening age to 20 may be beneficial. Nevertheless, it is important to recognize that the current screening target of age 35 already places a burden on health workers and peripheral health systems [72]. Any effort to lower the screening age as a strategy to address the early impact of environmental and lifestyle factors on diabetes must also consider these operational challenges.

Second, the study did not assess the impact at the individual level, so the associations between risk factors and outcomes should be interpreted cautiously. Another limitation is related to the health system and incomplete data. Notably, the study did not include data from Bangkok to represent urban settings, nor did it account for screenings and treatments conducted in private healthcare settings, which may limit the generalizability of the findings. Furthermore, the environmental parameters used in the study, such as temperature data obtained from satellite sources, may introduce reliability issues when compared to data collected by meteorological stations. Although a significant correlation (*r* = 0.55) was observed between the available station data and ERA5-Land data, the Intraclass Correlation Coefficient (ICC) for consistency was only 0.234, indicating weak agreement. Additionally, some provinces lacked air pollution stations, resulting in the use of data from various sources, which raises concerns about interrater reliability.

It is essential to note that the decline in PM2.5 levels annually (except at the later year) mainly reflects the addition of new monitoring stations in cleaner areas and may not reflect actual air quality improvement. Interpolation of missing data may overestimate earlier values, influencing associations. However, excluding new stations would reduce generalizability. Thus, we included all stations and interpolated missing values for completeness.

Future studies should focus on analyzing daily temperature variations and other environmental parameters using time-series data to explore lag effects and the short-term impact of environmental exposure on diabetes complications. Moreover, incorporating more granular, individual-level data could provide deeper insights into the specific effects of climate factors on diabetes outcomes and complications.

### Conclusion and recommendations

These findings highlight that climate-health responses are shaped by existing health systems and socio-economic contexts. Adaptation strategies must consider differences in coverage, infrastructure, and local norms. In Thailand, rising temperatures and PM2.5 levels affect diabetes screening, with persistent gaps in rural and disadvantaged areas. To strengthen resilience, diabetes screening should expand to community and home settings using community health workers and self-testing kits. Enhancing UHC and urban/private sector access is essential. Future research should explore short-term environmental impacts on diabetes outcomes to inform adaptive policies.

## Supplementary Information

Supplementary Material 1.

Supplementary Material 2.

Supplementary Material 3.

Supplementary Material 4.

Supplementary Material 5.

## Data Availability

The datasets generated and/or analysed during the current study are available in the Health Data Center Ministry of Public Health repository, but only accessible using Thailand internet network. Therefore, request for aggregated data should be addressed to corresponding author and with the permission from Ministry of Public Health of Thailand. The link for complete data and code to extract environmental data from ERA5 using google earth engine are available at https://figshare.com/s/bcbc5cb327f30cb20a74.
