# Supplementary material for: Ecological assessment of extreme temperature and fine particulate matter (pm2.5) impact on diabetes service and outcomes in Thailand
Source: BMC Public Health. 2025 Aug 15;25:2786. doi: 10.1186/s12889-025-24003-5 (PMC12355854; doi:10.1186/s12889-025-24003-5)
Supplement: Supplementary file 1 — Supplementary Material 1. [file 12889_2025_24003_MOESM1_ESM.docx]

**Supplementary Table 1. Generalized Additive Model Predicting Diabetes Screening with 7 interactions**

| **Predictor** | **Estimate** | **SE** | **t/F** | **p** |
| --- | --- | --- | --- | --- |
| **Parametric Coefficients** |  |  |  |  |
| Intercept | 0 | 0 | - | - |
| Health Insurance (HI) | 1.205 | 0.164 | 7.331 | < .001 |
| Health Personnel Rate (ddnrate) | 0.112 | 0.327 | 0.344 | 0.731 |
| COVID-19 Indicator | -1.694 | 1.519 | -1.115 | 0.266 |
| Sex Ratio | -22.462 | 16.075 | -1.397 | 0.164 |
| **Smooth Terms** |  |  |  |  |
| Temperature (maxtemp) | - | - | F = 1.398 | 0.19 |
| Health Volunteer Rate (HV) | - | - | F = 2.890 | 0.003 |
| BMI Abnormality Rate (BMIabnorm) | - | - | F = 5.404 | < .001 |
| PM2.5 (PM) | - | - | F = 0.563 | 0.827 |
| Alcohol Consumption (alc) | - | - | F = 6.539 | < .001 |
| Smoking Prevalence (smoking) | - | - | F = 16.632 | < .001 |
| Percentage of Community Health Funds (percentCH) | - | - | F = 1.699 | 0.091 |
| **Interaction Terms (Tensor Products)** |  |  |  |  |
| Temperature × Health Insurance (HI) | - | - | F = 13.052 | < .001 |
| Temperature × Health Volunteer Rate | - | - | F = 0.000 | 0.577 |
| PM2.5 × Health Insurance (HI) | - | - | F = 0.206 | 0.04 |
| PM2.5 × Health Volunteer Rate | - | - | F = 0.053 | 0.145 |
| Temperature × BMI Abnormality | - | - | F = 0.016 | 0.251 |
| Temperature × Community Health Funds | - | - | F = 0.056 | 0.151 |
| PM2.5 × Community Health Funds | - | - | F = 0.146 | 0.065 |
| **Spatial Smooth** |  |  |  |  |
| Latitude × Longitude | - | - | F = 1.860 | 0.028 |
|  |  |  |  |  |
| Deviance explained = 80%, Adjusted R² = .725, REML = 857.46, n = 304. | | | |  |
