# Supplementary material for: Ecological assessment of extreme temperature and fine particulate matter (pm2.5) impact on diabetes service and outcomes in Thailand
Source: BMC Public Health. 2025 Aug 15;25:2786. doi: 10.1186/s12889-025-24003-5 (PMC12355854; doi:10.1186/s12889-025-24003-5)
Supplement: Supplementary file 2 — Supplementary Material 2. [file 12889_2025_24003_MOESM2_ESM.docx]

**Supplementary Table 2. Additional analysis from google community data**

|  | Maximum Temperature | PM2.5 | Mobility |
| --- | --- | --- | --- |
| Jan-22 | 33.66 | 12.73 | 16.77 |
| Feb-22 | 33.68 | 10.59 | 30.03 |
| Mar-22 | 35.85 | 11.36 | 20.51 |
| Apr-22 | 37.33 | 12.61 | 21.77 |
| May-22 | 34 | 5.73 | 21.64 |
| un-22 | 34.38 | 4.16 | 21.87 |
| Jul-22 | 33.97 | 4.21 | 22.9 |
| Aug-22 | 33.07 | 4.19 | 26.48 |
| Sep-22 | 33.14 | 4.63 | 36.73 |
