# Supplementary material for: Ecological assessment of extreme temperature and fine particulate matter (pm2.5) impact on diabetes service and outcomes in Thailand
Source: BMC Public Health. 2025 Aug 15;25:2786. doi: 10.1186/s12889-025-24003-5 (PMC12355854; doi:10.1186/s12889-025-24003-5)
Supplement: Supplementary file 3 — Supplementary Material 3. [file 12889_2025_24003_MOESM3_ESM.docx]

The first supplementary table considers potential interactions between environmental factors, health system factors and socioeconomic factors. These are potential interactions that could be integrated into the model.

**1. Maximum temperature and health insurance coverage**

Why? People with better health insurance might access care even in harsh environmental conditions.
Relevance: High policy leverage—health insurance is a system-level intervention that can buffer environmental stress.

2. **Maximum temperature and health volunteer rate**

Why? Health volunteers can reduce barriers caused by extreme heat, e.g., home visits, local support. But extreme weather may introduce reluctancy to serve as health volunteers
Relevance: Community resilience strategy—empowering local actors to bridge access gaps during heatwaves.

3. **PM 2.5 and health insurance coverage**

Why? Air pollution might deter people from seeking care, but insurance could reduce cost-related hesitancy.
Relevance: System-environment interface—insurance mitigates the access drop-off during pollution spikes.

4. **PM 2.5 and health volunteer rate**

Why? Health volunteers might sustain service delivery during air pollution episodes via outreach or information.
Relevance: Community-level buffer—volunteers ensure continuity of services despite environmental disruptions.

5. **Maximum temperature and proportion of people with higher BMI**

Why? People with higher BMI may be more vulnerable to heat (e.g., mobility, comorbidities), affecting diabetes screening and service in areas with a higher proportion of obese and overweight people.
Relevance: Clinical vulnerability—tailoring interventions to high-risk populations in extreme temperatures.

6. **Maximum temperature and % Accredited Health Center**

Why? Accredited health centres likely have better facilities to maintain services during hot days (cooling, resources).
Relevance: Facility-level adaptation—evidence for investing in health center capacity to counter climate impacts.

7. **PM2.5 and % Accredited Health Center**

Why? Similarly, better-accredited centers might be more capable of operating during high pollution days (air filtration, protocols).
Relevance: Infrastructure resilience—supporting the case for quality improvements in health facilities as environmental adaptation.

**Supplementary Table 1. Generalized Additive Model Predicting Diabetes Screening with 7 interactions**

| **Predictor** | **Estimate** | **SE** | **t/F** | **p** |
| --- | --- | --- | --- | --- |
| **Parametric Coefficients** |  |  |  |  |
| Intercept | 0 | 0 | - | - |
| Health Insurance (HI) | 1.205 | 0.164 | 7.331 | < .001 |
| Health Personnel Rate (ddnrate) | 0.112 | 0.327 | 0.344 | 0.731 |
| COVID-19 Indicator | -1.694 | 1.519 | -1.115 | 0.266 |
| Sex Ratio | -22.462 | 16.075 | -1.397 | 0.164 |
| **Smooth Terms** |  |  |  |  |
| Temperature (maxtemp) | - | - | F = 1.398 | 0.19 |
| Health Volunteer Rate (HV) | - | - | F = 2.890 | 0.003 |
| BMI Abnormality Rate (BMIabnorm) | - | - | F = 5.404 | < .001 |
| PM2.5 (PM) | - | - | F = 0.563 | 0.827 |
| Alcohol Consumption (alc) | - | - | F = 6.539 | < .001 |
| Smoking Prevalence (smoking) | - | - | F = 16.632 | < .001 |
| Percentage of Community Health Funds (percentCH) | - | - | F = 1.699 | 0.091 |
| **Interaction Terms (Tensor Products)** |  |  |  |  |
| Temperature × Health Insurance (HI) | - | - | F = 13.052 | < .001 |
| Temperature × Health Volunteer Rate | - | - | F = 0.000 | 0.577 |
| PM2.5 × Health Insurance (HI) | - | - | F = 0.206 | 0.04 |
| PM2.5 × Health Volunteer Rate | - | - | F = 0.053 | 0.145 |
| Temperature × BMI Abnormality | - | - | F = 0.016 | 0.251 |
| Temperature × Community Health Funds | - | - | F = 0.056 | 0.151 |
| PM2.5 × Community Health Funds | - | - | F = 0.146 | 0.065 |
| **Spatial Smooth** |  |  |  |  |
| Latitude × Longitude | - | - | F = 1.860 | 0.028 |
|  |  |  |  |  |
| Deviance explained = 80%, Adjusted R² = .725, REML = 857.46, n = 304. | | | |  |

As shown in Supplementary Table 1, the generalized additive model explained a substantial proportion of the variance in diabetes screening rates (Deviance explained = 80%, Adjusted R² = .725, REML = 857.46). Among the parametric predictors, **health insurance coverage (HI)** was significantly associated with higher screening rates (β = 1.205, SE = 0.164, t = 7.331, p < .001), highlighting the critical role of financial protection in facilitating access to care. Other parametric variables, including health personnel rate, COVID-19 indicator, and sex ratio, did not show significant associations.

Several **non-linear smooth effects** were identified. Notably, the **health volunteer rate (HV)** (F = 2.890, p = .003), **Proportion of People with higher BMI (BMIabnorm)** (F = 5.404, p < .001), **Proportion of Alcohol Drinker(alc)** (F = 6.539, p < .001), and **Proportion of Active Smokers(smoking)** (F = 16.632, p < .001) were all significantly associated with screening rates. These results emphasize the importance of community-level factors and health behaviors in shaping service utilization patterns.

Regarding **environment-health system interactions**, several key findings emerged:

1. **Maximum temperature × health insurance coverage**
   The interaction between maximum temperature and health insurance coverage was significantly linked to diabetes screening rates (F = 13.052, p < .001). Specifically, areas with **higher insurance coverage (above median)** experienced a **decline in screening rates as temperatures increased**. In contrast, in areas with **lower insurance coverage (at or below the 25th percentile)**, screening rates **increased with rising temperatures**. This suggests that higher insurance coverage areas might face system-level barriers or behavioral changes under heat stress, leading to reduced service utilization, while in low-coverage areas, temperature might not deter people from seeking screening, possibly due to different socio-economic or healthcare access dynamics.
2. Although the interaction between **maximum temperature × health volunteer rate** was not statistically significant (F = 0.000, p = .577), it reflects a potentially important community resilience strategy. Health volunteers can mitigate access barriers during heatwaves through localized support, albeit extreme temperatures may affect their service capacity.
3. The interaction between **PM2.5 × health insurance coverage** was statistically significant (F = 0.206, p = .040), indicating that insurance mitigates the negative impact of air pollution on healthcare access. This finding reinforces the system-environment interface perspective, where insurance reduces cost-related hesitancy during pollution episodes.
4. The interaction of **PM2.5 × health volunteer rate** was not significant (F = 0.053, p = .145), though conceptually relevant. Health volunteers might play a community-level buffering role, ensuring service continuity despite environmental disruptions.
5. The interaction of **maximum temperature × Proportion of People with higher BMI** was not significant (F = 0.016, p = .251), but highlights an important clinical vulnerability. Populations with higher BMI may be more affected by extreme heat, suggesting the need for tailored interventions in these groups.
6. The interaction between **maximum temperature × percentage of accredited health centers** did not reach significance (F = 0.056, p = .151), yet it reflects the potential facility-level adaptations necessary to maintain services during extreme heat events.
7. Similarly, **PM2.5 × percentage of accredited health centers** approached significance (F = 0.146, p = .065), suggesting a trend where better infrastructure resilience in accredited centers could help sustain service provision during high pollution days.

Lastly, the **spatial smooth term (Latitude × Longitude)** was significant (F = 1.860, p = .028), indicating spatial heterogeneity in diabetes screening rates across provinces, warranting region-specific strategies.


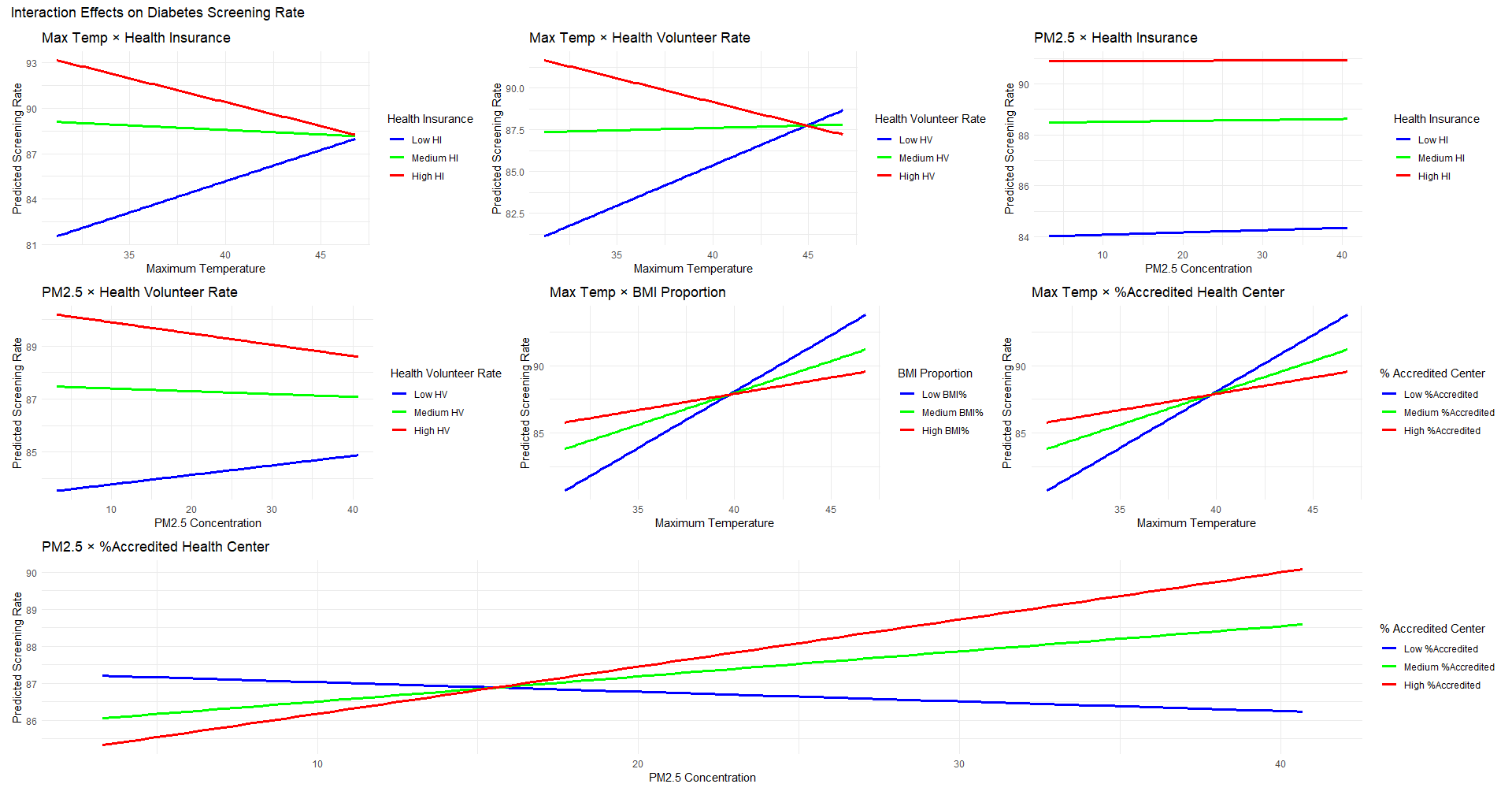


Further interaction analysis shows significant interaction between health insurance coverage and both maximum temperature (p<0.001), where areas with higher insurance coverage (above median) experienced a decline in screening rates as temperatures increased. In contrast, in areas with lower insurance coverage (at or below the 25th percentile), screening rates increased with rising temperatures
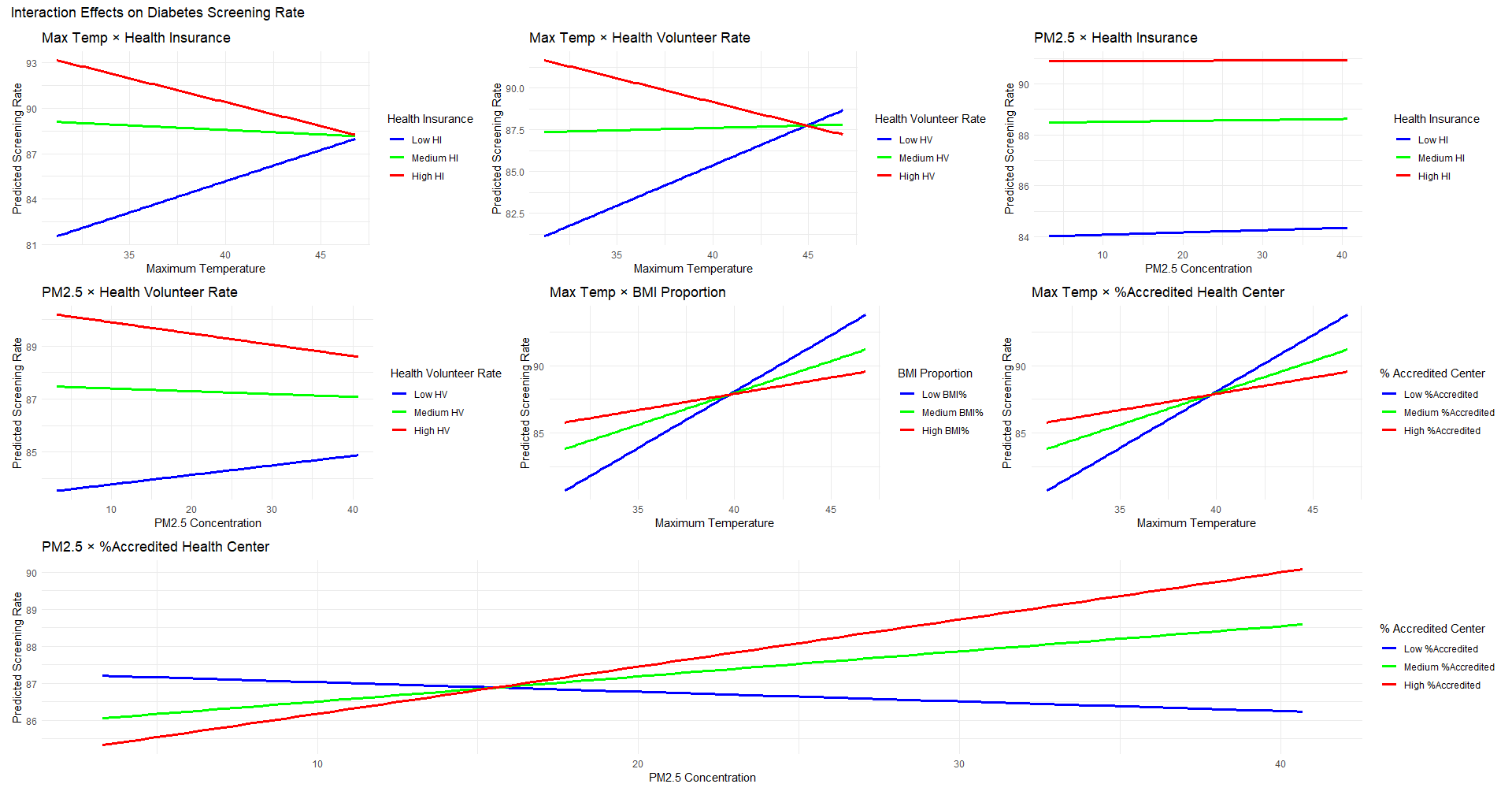


In contrast, The interaction between PM2.5 and health insurance coverage was significantly linked to diabetes screening rates. All quartiles of Health Insurance (HI) coverage shows an increase of diabetes screening following the increase of PM 2.5 value, and the lower HI shows the drastical increase although lower than other higher quartiles


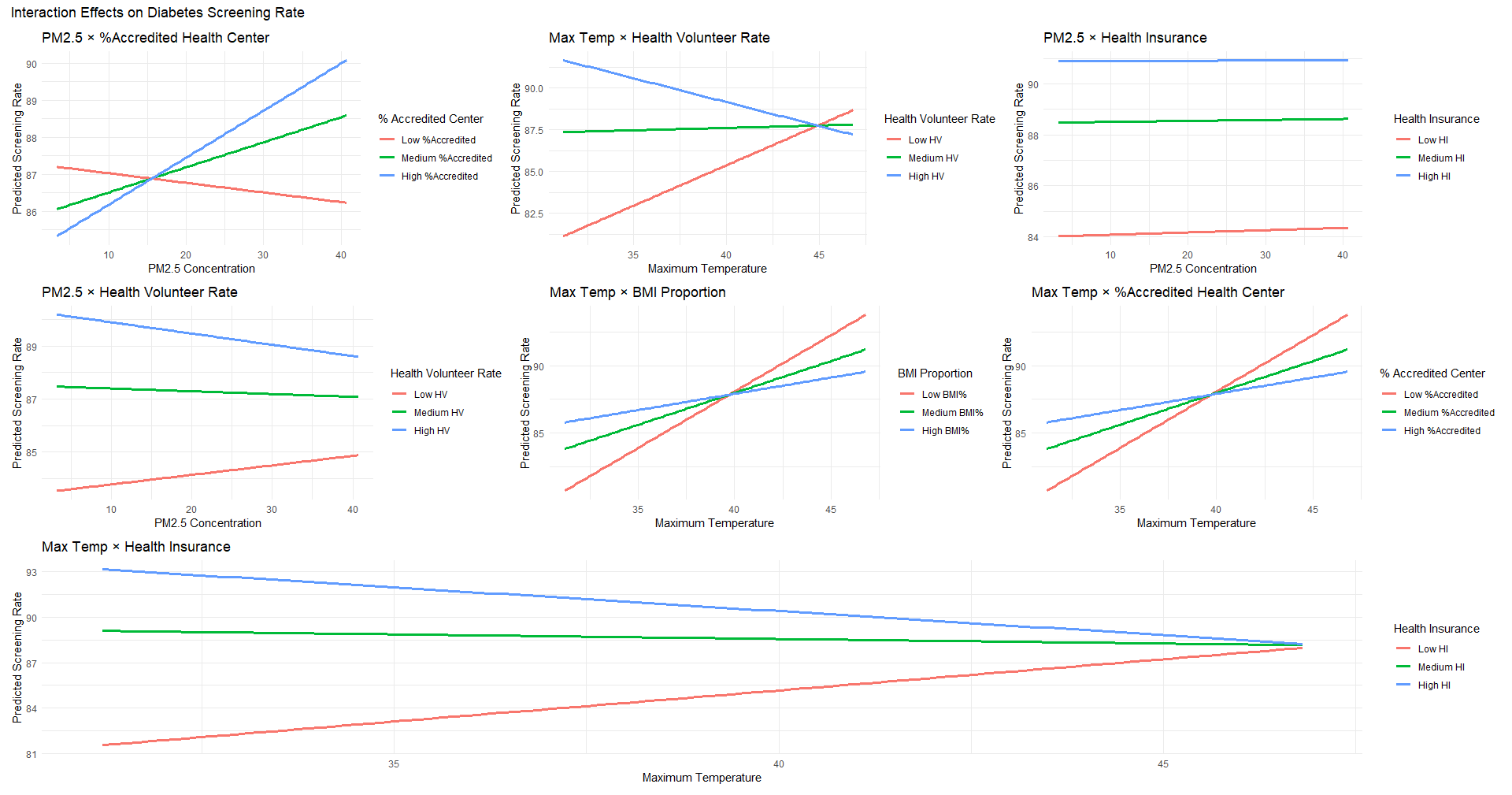


the interaction between PM2.5 and the percentage of accredited health centers revealed a different trend: areas with a lower percentage of accredited centers experienced decreased screening rates as PM2.5 levels increased, while areas in the upper percentile showed a positive trend.

**Supplementary Table 2. Additional analysis from google community data**

|  | Maximum Temperature | PM2.5 | Mobility |
| --- | --- | --- | --- |
| Jan-22 | 33.66 | 12.73 | 16.77 |
| Feb-22 | 33.68 | 10.59 | 30.03 |
| Mar-22 | 35.85 | 11.36 | 20.51 |
| Apr-22 | 37.33 | 12.61 | 21.77 |
| May-22 | 34 | 5.73 | 21.64 |
| un-22 | 34.38 | 4.16 | 21.87 |
| Jul-22 | 33.97 | 4.21 | 22.9 |
| Aug-22 | 33.07 | 4.19 | 26.48 |
| Sep-22 | 33.14 | 4.63 | 36.73 |

Supplementary Material 1


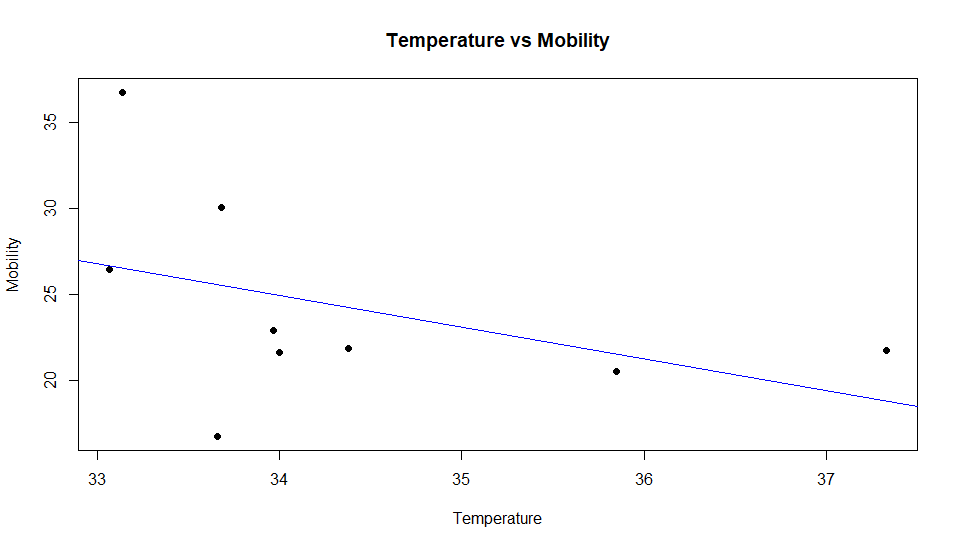

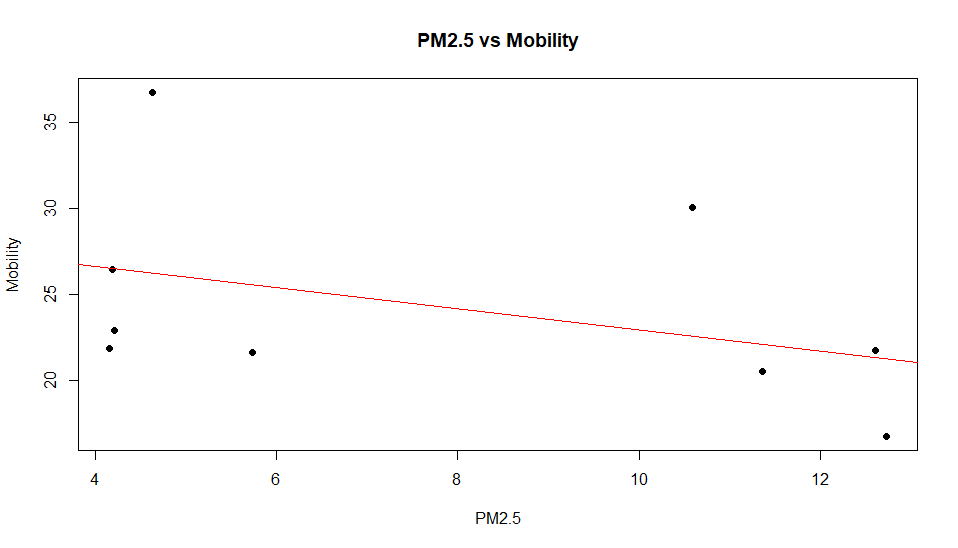


Supplementary Material 2


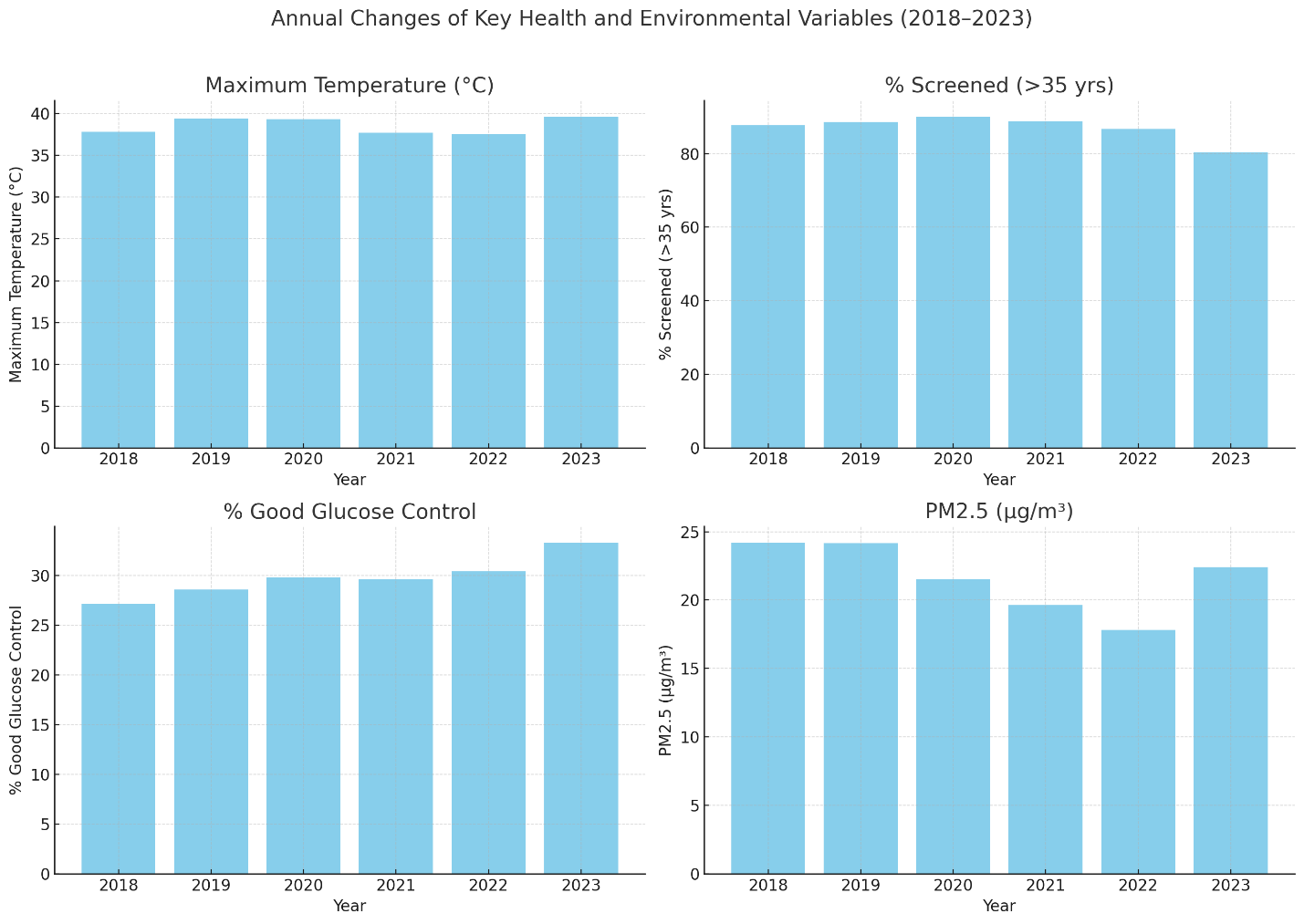


This graph presents the year-by-year changes in selected health and environmental indicators related to diabetes screening and control between 2018 and 2023. The average **maximum temperature** showed some fluctuation over the years, peaking in 2019 (39.39°C) and 2023 (39.63°C), with a dip observed in 2021 (37.73°C). The **percentage of the target population screened** for diabetes (individuals aged >35 years) remained relatively high from 2018 to 2021 (ranging from 87.69% to 90.03%), but experienced a noticeable decline in 2023 (80.31%). The **proportion of patients achieving good blood glucose control** showed a steady annual increase, rising from 27.17% in 2018 to 33.29% in 2023, indicating overall improvement in diabetes management outcomes. Meanwhile, the **annual average PM2.5 levels** decreased from 24.18 µg/m³ in 2018 to a low of 17.81 µg/m³ in 2022, before slightly rising again in 2023 to 22.40 µg/m³. These trends may reflect changes in environmental conditions, public health efforts, and healthcare service performance over time.
