# Supplementary figures and images for: Ecological assessment of extreme temperature and fine particulate matter (pm2.5) impact on diabetes service and outcomes in Thailand
Source: BMC Public Health. 2025 Aug 15;25:2786. doi: 10.1186/s12889-025-24003-5 (PMC12355854; doi:10.1186/s12889-025-24003-5)

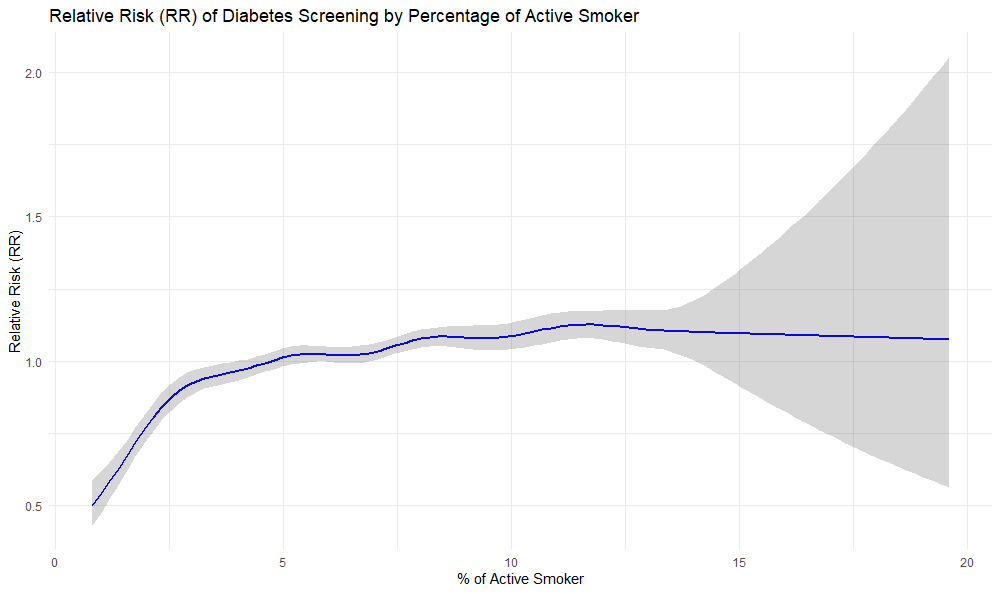

Supplement: Supplementary file 4 — Supplementary Material 4. [file 12889_2025_24003_MOESM4_ESM.png]

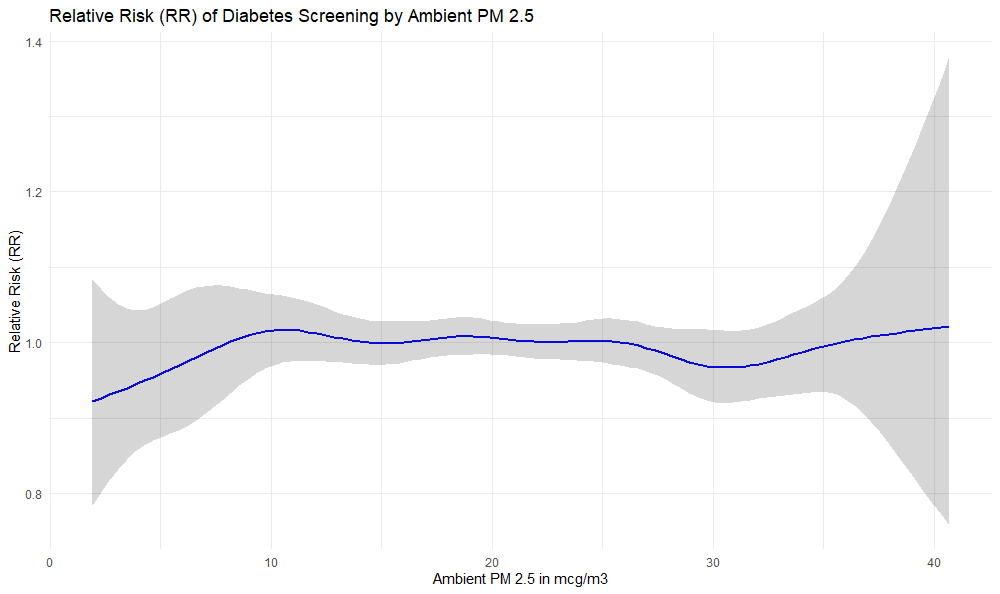

Supplement: Supplementary file 5 — Supplementary Material 5. [file 12889_2025_24003_MOESM5_ESM.png]
